# Inflamed Colon and Pericardium: A Rare Combination of Colitis and Recurrent Pericarditis

**DOI:** 10.1016/j.jaccas.2021.04.009

**Published:** 2021-05-26

**Authors:** Ashwin K. Kumar, Muhammad M. Furqan, Abdullah Yesilyaprak, Beni R. Verma, Mohamed Gad, Hassan M. Lak, Dakshin Gangadharamurthy, Reza Reyaldeen, Allan L. Klein

**Affiliations:** a Center for the Diagnosis and Treatment of Pericardial Diseases, Section of Cardiovascular Imaging, Department of Cardiovascular Medicine, Heart, Vascular, and Thoracic Institute, Cleveland Clinic, Cleveland, Ohio, USA; b Department of Cardiology, Magnolia Regional Health Center, Cornith, Mississippi, USA

**Keywords:** cardiac magnetic resonance imaging, multimodality imaging, recurrent pericarditis, rilonacept, ulcerative colitis, CMR, cardiac magnetic resonance imaging, DHE, delayed hyperenhancement, ECG, electrocardiogram, IL, interleukin, STIR, short-tau inversion recovery, UC, ulcerative colitis

## Abstract

A 29-year-old woman with severe ulcerative colitis presented with complicated recurrent pericarditis. Cardiac magnetic resonance imaging showed improvement in pericardial inflammation with a prolonged course of anti-inflammatory therapy, but she developed several relapses on biologics. Rilonacept (newer interleukin-1 antagonist), disease-modifying antirheumatic drugs, and pericardiectomy may be considered in such patients. (**Level of Difficulty: Intermediate.**)

## Central Illustration

**Figure:**
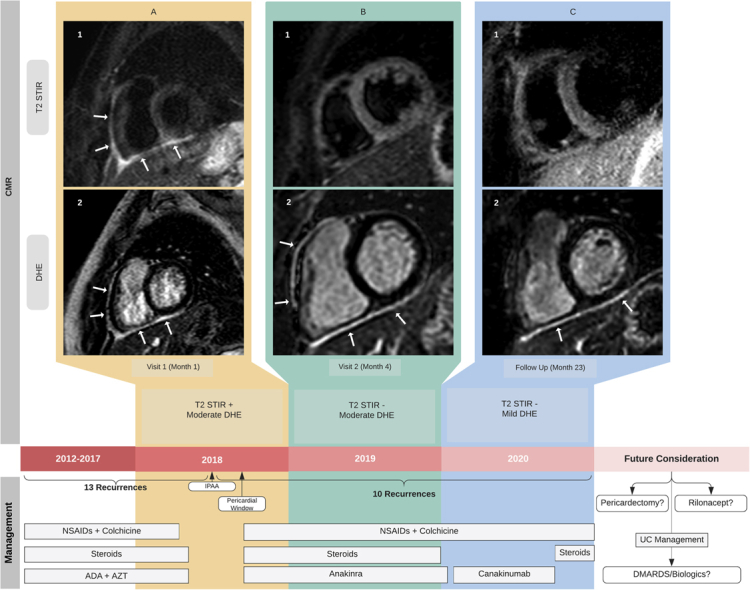

## History of Presentation

A 29-year-old woman with recurrent pericarditis presented with pleuritic chest pain, dyspnea, and fatigue in the fall of 2018. She underwent ileal pouch–anal anastomosis after failing medical management of ulcerative colitis (UC) in the summer of 2018. One month after the ileal pouch–anal anastomosis, she was admitted to an outside hospital for a large pericardial effusion and underwent a pericardial window. She was given naproxen (500 mg a day), colchicine (0.6 mg a day), and prednisone (20 mg a day) following the pericardial drainage.

**Learning Objectives**

• To identify recurrent pericarditis in the differential for patients with underlying ulcerative colitis and repeated episodes of chest pain.
• To be able to use serial cardiac magnetic resonance imaging in managing complicated recurrent pericarditis cases.
• To understand the management of recurrent pericarditis patients experiencing relapses during the treatment with multiple anti-inflammatory medications including biologics.

## Past Medical History

She has a history of recurrent pericarditis refractory to nonsteroidal anti-inflammatory drugs, colchicine, and steroids, and UC intractable to azathioprine, steroids, adalimumab, and infliximab.

## Differential Diagnosis

Differential diagnosis included pericarditis, acute coronary syndrome, congestive heart failure, and pulmonary embolism.

## Investigations

**Figure 1:**
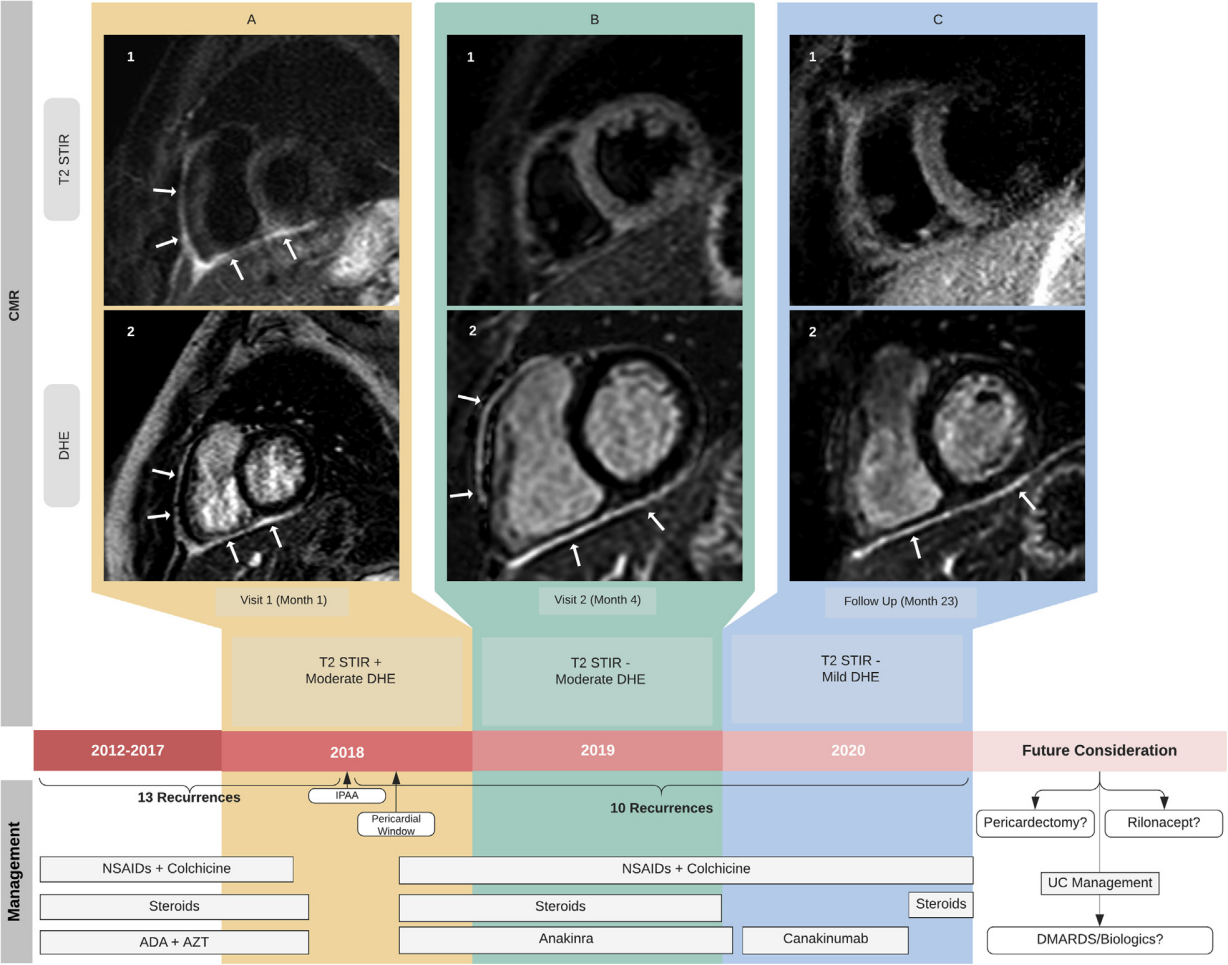
Timeline of Disease With Corresponding CMR Images **(A1)** Initial CMR demonstrating evidence of increased pericardial signal intensity **(arrows)** along the anterior and diaphragmatic aspects on T2 STIR imaging, suggestive of active pericardial edema/inflammation. **(A2)** Pericardial hyperintensity in the same areas on late gadolinium sequences **(arrows)**, overall consistent with acute/sub-acute pericarditis. **(B1)** No evidence of increased pericardial signal intensity on T2 STIR imaging to suggest pericardial edema. **(B2)** Persistent moderate pericardial hyperenhancement on late gadolinium sequences **(arrows)**, indicative of sub-acute/chronic pericarditis. **(C1)** No significant increased pericardial signal intensity on T2 STIR imaging to suggest pericardial edema. **(C2)** Mild pericardial hyperenhancement on late gadolinium sequences **(arrows)***,* predominantly at the diaphragmatic aspect suggestive of ongoing, chronic pericarditis. ADA = adaliumumab; AZT = azathioprine; CMR = cardiac magnetic resonance imaging; DHE = delayed hyperenhancement; DMARD = disease-modifying anti-rheumatic drugs; IPAA = ileal pouch–anal anastomosis; NSAID = non-steroidal anti-inflammatory drug; STIR = short-tau inversion recovery; UC = ulcerative colitis.

On presentation, physical examination revealed normal S1 and S2, with no murmurs, rubs, or gallops appreciated. Electrocardiogram (ECG) and serial troponins were normal. She had a slightly elevated sedimentation rate (24 mm/h), but C-reactive protein was normal (0.1 mg/dL). Interferon-gamma release assay for tuberculosis and anti-nuclear antibody were negative. No effusion or pericardial thickening was seen on echocardiography. Cardiac magnetic resonance imaging (CMR) of the pericardium showed increased T2 short-tau inversion recovery (STIR) indicative of edema and moderate pericardial delayed hyperenhancement (DHE) indicative of inflammation consistent with active pericarditis (Figure 1A).

## Management (medical/interventions)

She had tapered her prednisone slowly and was on dual anti-inflammatory therapy (naproxen and colchicine). Because her symptoms recurred and CMR findings confirmed pericardial edema and inflammation, her prednisone (20 mg) was restarted, and anakinra was also added to the treatment regimen. Her colchicine dosage was increased to 0.6 mg twice a day and naproxen (500 mg) was maintained. A second CMR after 4 months showed no signal intensity on T2 STIR with moderate DHE (Figure 1B). Her prednisone was tapered off in a year, but she had 6 relapses of chest pain during this period. She also developed an intractable cough on anakinra 100 mg subcutaneous daily, which was changed to canakinumab 150 mg subcutaneous every 8 weeks. She had a CMR at month 23 that showed mild DHE (Figure 1C). Serial echocardiography demonstrated no pericardial effusion or other significant findings. The erythrocyte sedimentation rate remained slightly elevated, but C-reactive protein was within normal limits (Table 1). She experienced several episodes of chest pain and multiple recurrent urinary tract infections while on canakinumab, and it was eventually discontinued. Prednisone (20 mg) was restarted, and she was advised to maintain regular follow-up with cardiology and rheumatology.

**Table 1:** Trend of Inflammatory Markers

Inflammatory Markers	Winter 2018	Spring 2019	Summer 2019	Summer 2020	Autumn 2020
ESR, mm/h∗	24	25	16	27	37
CRP, mg/dl†	0.1	0.2	0.1	0.3	0.3

CRP was normal, and ESR remained slightly increased.

CRP = C-reactive protein; ESR = erythrocyte sedimentation rate.

∗ Normal value <15 mm/h;

† normal value <0.9 mg/dl.

## Discussion

Cardiac involvement (myocarditis, myopericarditis, and pericarditis) is rare in UC, and pericarditis is specifically found only in around 0.23% of UC patients (1). However, the prevalence of recurrent pericarditis in UC is unknown. This patient represents a complex case of recurrent pericarditis in the setting of severe UC. The pathophysiology of UC is multifactorial and linked to an increase in expression of Toll-like receptor-4 leading to activation of nuclear factor kappa B (NF-kB). NF-kB regulates macrophage production of proinflammatory cytokines (tumor necrosis factor-alpha, and interleukin [IL]-1, and IL-6) (2). Patients with UC also have a defective Th2 helper cell response, which results in the up-regulation of various other cytokines (IL-5 and IL-13). This dysregulation in the immune system and increased activity of cytokines lead to tissue injury in UC (3).

Clinical diagnosis of pericarditis requires that patients fulfill 2 of 4 major criteria, including pleuritic chest pain, diffuse ST-segment elevation/PR depression on ECG, pericardial rub, and presence of effusion on imaging. Furthermore, patients with recurrence may not have all the characteristic findings of pericarditis (4). Our patient experienced varying symptoms during multiple episodes and a prolonged course of pericarditis making management difficult. She was also on several anti-inflammatory drugs, which could explain the near-normal inflammatory markers and nondiagnostic echocardiography.

A subset of patients with pericarditis requires further imaging with CMR. CMR has developed into an important tool for assessing severity and response to treatment in recurrent pericarditis. CMR findings of pericarditis include enhancement of the thickened pericardium on T1-weighted images and increased intensity of the pericardial tissue seen on T2-weighted images (5). Increased intensity upon gadolinium administration, a phenomenon referred to as DHE, is also seen (5). CMR findings of pericardial edema on DHE have a 99% specificity for the diagnosis of pericarditis (4). However, the use of CMR is usually limited due to time, administration of contrast material, and financial cost. Therefore, CMR is done in complicated cases and in patients with persistent pleuritic chest pain with a high suspicion of pericarditis where an ECG and echocardiogram are nondiagnostic (5,6). This patient had positive CMR findings for pericardial edema (T2 STIR) and inflammation (DHE) on presentation representative of subacute pericarditis (Figure 1A).

Mainstays of treatment of recurrent pericarditis involve nonsteroidal anti-inflammatory drugs and colchicine. Low-dose steroids may be used in patients resistant to first-line therapy. Biologics, notably IL-1 blockers such as anakinra (IL-1α and IL-1β receptor antagonist) and canakinumab (IL-1β monoclonal antibody), are indicated in patients developing dependence on second-line agents (6,7). Subsequent CMR images (Figure 1B) showed resolution of edema after 4 months, but it took longer to see any significant improvement in the inflammation on DHE (Figure 1C). She was given biologics and was able to taper steroids. However, she experienced multiple recurrences even with the biologics and also experienced side effects to both anakinra and canakinumab. Therefore, her biologics were discontinued, and she was advised to continue prednisone (20 mg).

To our knowledge, this is one of the first reports of a severe UC patient with pericarditis resistant to multiple agents. Despite numerous attempts to optimize medical management for our patient, she was unable to achieve complete remission. It is possible that the underlying autoimmune pathology of complicated ulcerative colitis plays a role in ongoing inflammation and resistance to multiple therapies.

## Follow-Up

The patient developed another recurrence 3 months after the discontinuation of canakinumab, and her prednisone dose was increased to 40 mg. The next management steps under discussion are rilonacept (320 mg subcutaneous load followed by 160 mg per week) (IL-1α and -1β antagonist) or radical pericardiectomy for definitive pericardial symptom resolution (8). Additionally, adding disease-modifying antirheumatic agents to her steroid regimen may be beneficial for her underlying inflammatory bowel disease.

## Conclusions

Management of complicated recurrent pericarditis with concomitant UC is challenging, and patients may develop resistance to multiple anti-inflammatory medications and relapses of chest pain on biologics. A severe case of underlying autoimmune disease in recurrent pericarditis patients requires a long course of medications, and serial CMR can help to assess treatment response.

## Funding Support and Author Disclosures

Dr Klein has received research funding from Kiniksa Pharmaceuticals, Ltd; and has served on scientific advisory boards for Kiniksa Pharmaceuticals, Ltd, Swedish Orphan Biovitrum AB, and Pfizer, Inc. All other authors have reported that they have no relationships relevant to the contents of this paper to disclose.
